# An Entropy Approach to Interdependent Human–Machine Teams

**DOI:** 10.3390/e27020176

**Published:** 2025-02-07

**Authors:** William Lawless, Ira S. Moskowitz

**Affiliations:** 1 Department of Mathematics and Psychology, Paine College, Augusta, GA 30901, USA; 2 Information Technology Division, Naval Research Laboratory, Washington, DC 20375, USA; ira.s.moskowitz.civ@us.navy.mil

***Overview of recent developments in the field***. Recently, Bengio et al. [1] proposed in the journal *Science* that AI is changing rapidly, and it may pose

“risks that include large-scale social harms, malicious uses, and an irreversible loss of human control over autonomous AI systems.”

Unlike leaders who may not counter this threat, we have discovered that the self-organization of autonomous humans conveyed by interdependence is a resource that counters threats [2]. Further, Cummings [3] reported that the best teams of human scientists worked in states of maximum interdependence. But how can we generalize from human teams to human–machine teams?

We began this Special Issue in *Entropy* by recognizing that generalizing interdependence to human–machine teams “… presents … [a] series of challenges … [with] more questions than answers …” Manipulating states of interdependence in a team of humans is considered too difficult to manage in the laboratory (Jones, 1998, p. 33; in [4]). As an example of generalizing, inside of a machine’s team, if one machine’s process, *A*, is dependent on another machine’s process, *B*, machine *B’s* process is dependent on a third machine’s process, *C*, and the results are dependent on *A*, *B* and *C*, non-verbal interdependence performs well (e.g., chem-labs developing new laser materials, in [5]), but does so by ignoring cognitive beliefs about the situation, context, or world at large.

***The gaps in existing knowledge and how this Special Issue addressed them***. Our goal to achieve interdependent cognition and behavior is not as far along (viz., where teammates both speak and react physically to each other); for example, from the *Wall Street Journal* [6], robots cannot yet manage other robots or each other:

“Some traditional warehouse roles have proved too difficult for Amazon to fully automate … Humans [but not robots] can easily look into a storage container packed full of goods, identify a particular item and know how to pick it up and handle it …”

Still, the development of generative AI is rapid. In this Special Issue, our contributors addressed some of the following gaps:

1. The first article, by Abdo’s team, explored collaboration and strategies in cyber competition with agent-based models (ABMs).

2. In two articles, Boris Galitsky addressed deep learning neural nets to improve explanations and question answering by large language models (LLMs).

3. Scheuerman’s team addressed uncertainty and data drift with iterative feedback in human–machine teams.

4. Moskowitz’s team explored optimal decision making for multi-agent systems, finding that the way in which agents are combined influences channel capacity.

5. Russell’s team combined qualitative and quantitative health data and human expertise to find that the combination increased precision.

6. Santos’ team applied inverse reinforcement learning to command styles and interference in swarms, finding that heterogeneous scenarios worked best.

7. Lawless applied complementary cognition and behavior in human–machine teams to find energy-entropy trade-offs in team decisions.

8. Finally, Mylrea’s team crafted a trust framework and notional data to underscore the need for ethical safeguards in AI.

***Future research that should be considered***. The problem with machines and robotics is that while they are more capable, they are not yet competent with interdependent language and behavior [7]. A contributing factor to this problem is that classical social science has not been able to establish a valid process to construct and test the reproducibility of concepts. As examples of scales that have been found to be invalid, the claims made for self-esteem could not be reproduced in 2005 [8]; implicit racism in 2009 [9]; ego-depletion in 2016 [10]; and the honesty scale in 2021.^1^ The reproducibility crisis was identified formally in 2015 [11], leading to a plan to strengthen the tests for conceptual scales by, among other things, the preregistration of measures and analyses supporting claims before a reproducibility experiment was run. However, the test of the plan itself was retracted after the editors of *Nature* lost confidence based on criticisms raised about the results then being produced.^2^

Moreover, the National Academy of Sciences [12] reported that unraveling interdependent effects may not even be possible:

The “performance of a team is not decomposable to, or an aggregation of, individual performances …”

These problems with cognitive science suggest a loss of information within the interaction, acting like quantum superposition [7]. However, the opposite problem yet with the same result occurs with behaviorism (e.g., [13]). From a different perspective and also with the same result, logic applied to collective decision making while operating in the open has also failed [14]. These problems with social science, logic and the academy all support the conclusions drawn by Jones [4].

Alternatively, the assembly theory of alien life [15] avoids the complications of interdependence by counting successful assemblies: the greater the number, the more likely that life is involved. Building on assembly theory’s idea of observable effects, by focusing on the observable results of interdependence in a team, we have found that entropy is key: by managing to produce a low entropy in a team’s structure, the team can maximize its performance by producing the maximum entropy in its output [2].

In sum, future research must mathematically integrate and model the effects of interdependence for both cognition/behavior and human–machine teams by

1. Deriving a theory of integrated cognition/behavior (viz., “words alone do not equal reality” [16]) that addresses all risks posed by AI to humans.

2. Assuming “words alone do not equal reality” means that the tools of generative AI (e.g., tensors, LLMs and machine learning) used to model “reality” are formed from separable entities that generate independent and identically distributed random variable (i.i.d.) data, but that cannot recreate whatever social interactions are captured with the data [16]. This situation distinguishes large language models from quantum superposition and entanglement [17]:

“The term tensor is one that comes up a lot in the context of machine learning … e.g., [a tensor product is] a matrix multiplication or a convolution, performed on any number of input tensors and resulting in a single output tensor.”

However, if we can write a quantum state as a tensor product of two quantum states, then we say that the states are not entangled [18]. This discussion helps us to understand why separable tensor products and, in general, i.i.d. data [16], cannot recreate whatever social interactions have been captured [2]. Why? Words account for only 7 percent of communication [19]; moreover, we do not need language to think [20]:

“Language is a defining characteristic of our species, but the function, or functions, that it serves has been debated for centuries. Here, we … argue that in modern humans, language is a tool for communication … [not] for thinking.”

From this discussion, to match the claim by the National Academy of Sciences regarding the decomposition of teams, we conclude that interdependence is quantum-like [2] and cannot be represented by a tensor product [7].

3. Including negative and positive emotion. We have proposed a mathematics of state dependency [21] to model interdependence for human–machine teams [7]. In this model, emotional distress increases entropy in a team’s structure, a trade-off diverting available energy from a team’s maximum entropy production, reducing a team’s performance [2]. Generalized to a model of society with quantum-like coupled harmonic oscillators could fulfill Lewin’s vision of an interdependent whole [22], such as a human–machine team, organization or system. There, under the uncertainty caused by free choice (e.g., the failure rate of corporate mergers is about 50%; in [23], exemplified by the recent failure of Walgreens’ merger; in [24]), political compromise [25] and innovation might be combined into an index that differentiates evolvable, autonomous, and observable self-organized assemblies ([15]; e.g., [26]) of interdependence randomly seeking the positive emotion of “animal spirits” [27]:

“… if the animal spirits are dimmed and spontaneous optimism falters, leaving us to depend on nothing but a mathematical expectation, enterprise will fade and die.”

## Notes

1 The honesty scale was retracted by the Editor of PNAS, Berenbaum, M.R. *Proc. Natl. Acad. Sci. USA* **2021**, *118*, e2115397118.

2 See the retraction at: Protzko, J.; Krosnick, J.; Nelson, L.; Nosek, B.A.; Axt, J.; Berent, M.; Buttrick, N.; DeBell, M.; Ebersole, C.R.; Lundmark, S.L.; et al. Retracted Article: High replicability of newly discovered social-behavioural findings is achievable. *Nature Hum. Behav.* **2024**, *8*, 311–319. https://doi.org/10.1038/s41562-023-01749-9.
